# Low CD10 mRNA Expression Identifies High-Risk Ductal Carcinoma In Situ (DCIS)

**DOI:** 10.1371/journal.pone.0012100

**Published:** 2010-08-10

**Authors:** Jérôme Toussaint, Virginie Durbecq, Sevilay Altintas, Valérie Doriath, Ghizlane Rouas, Marianne Paesmans, Philippe Bedard, Benjamin Haibe-Kains, Wiebren A. Tjalma, Denis Larsimont, Martine Piccart, Christos Sotiriou

**Affiliations:** 1 Breast Cancer Translational Research Laboratory JC Heuson, Jules Bordet Institute, Brussels, Belgium; 2 Medical Oncology Department, Jules Bordet Institute, Brussels, Belgium; 3 Medical Oncology Department, Antwerp University Hospital, Edegem, Belgium; University Medical Center Utrecht, Netherlands

## Abstract

**Purpose:**

Optimal management of breast ductal carcinoma *in situ* (DCIS) is controversial, and many patients are still overtreated. The local death of myoepithelial cells (MECs) is believed to be a pre-requisite to tumor invasion. We thus hypothesized that loss of CD10 expression, a MEC surface peptidase, would signify basement membrane disruption and confer increased risk of relapse in DCIS. The aim of our study was to retrospectively evaluate the prognostic value of CD10 in DCIS.

**Experimental Design:**

CD10 expression was evaluated by quantitative RT-PCR and immunohistochemistry using paraffin-embedded samples of normal breast tissue (n = 11); of morphologically normal ducts associated with DCIS (n = 10); and of DCIS without an invasive component (n = 154).

**Results:**

CD10 immunostaining was only observed in MECs in normal tissue and in DCIS. Normal tissue showed high mRNA expression levels of CD10, whereas DCIS showed a variable range. After a median follow-up of 6 years, DCIS with CD10 expression below the levels observed in normal tissue (71%) demonstrated a higher risk of local relapse (HR = 1.88; [95CI:1.30–2.70], p = 0.001) in univariate analysis. No relapse was observed in patients expressing high CD10 mRNA levels (29%) similar to the ones observed in normal tissue. In multivariate analysis including known prognostic factors, low CD10 mRNA expression remained significant (HR = 2.25; [95%CI:1.24–4.09], p = 0.008), as did the recently revised Van Nuys Prognostic Index (VNPI) score (HR = 2.03; [95%CI:1.23–3.35], p = 0.006).

**Conclusion:**

The decrease of CD10 expression in MECs is associated with a higher risk of relapse in DCIS; this knowledge has the potential to improve DCIS management.

## Introduction

With the widespread use of high quality screening mammography, the incidence of ductal carcinoma in situ (DCIS) has increased dramatically. DCIS now accounts for approximately 20% of all screening-detected breast cancer [1]. DCIS is pathologically described as the proliferation of malignant epithelial cells that have not invaded beyond the basement membrane. In reality, DCIS is a heterogeneous group of pre-invasive breast cancers with various patterns of morphology, expansion, and malignant potential.

The long-term prognosis of DCIS is excellent, with an expected overall 10-year survival rate that exceeds 95%, even in the absence of treatment [2]. However, 16% to 22% of women experience local relapse within 10 years following lumpectomy alone, and approximately one-half of these relapses are invasive breast cancer. Adjuvant radiotherapy reduces the 10-year risk of relapse to 7% to 9%, although it is associated with cutaneous toxicity and a small long-term risk of secondary neoplasms [3]–[5].

The Van Nuys Prognostic Index (VNPI) is a useful tool to evaluate the risk of local relapse. In the original VPNI [6] three independent histo-pathological parameters of relapse were considered: tumor size, margin width and pathological grade. Recently, the VNPI was revised to include the age of the patient at diagnosis [7]–[9]. The VNPI score is divided into three risk groups: low, intermediate and high. For low VPNI risk, lumpectomy alone is recommended, while patients with intermediate VPNI risk are strongly recommended to receive adjuvant radiotherapy following lumpectomy, and high-risk patients are advised to undergo mastectomy. However, many patients in the intermediate and high-risk categories would not relapse with lumpectomy alone and are overtreated with adjuvant radiotherapy or mastectomy. Therefore, additional prognostic biomarkers are needed to improve decision-making following resection of DCIS.

There is growing evidence that alterations in the tumor microenvironment might underlie local relapse in DCIS. Myoepithelial cells (MECs) that surround mammary ducts and lobular acini are involved in mammary gland homeostasis and prevent breast cancer progression. MECs maintain the basement membrane, forming a physical barrier between epithelial cells and the surrounding stroma, which blocks tumor cell invasion. MECs also secrete paracrine mediators that inhibit tumor growth, invasion and angiogenesis [10]–[12]. Two hypotheses (models) should explain the transition from *in situ* to invasive carcinoma: the “escape” and the “release” models. In the escape model, the tumor epithelial cells disrupt the MEC layer, degrade the basement membrane, and migrate into the stroma, whereas in the “release” model, the MECs disappear and the basement membrane is disrupted at sites coinciding with areas of leukocytic infiltration and accumulation of myofibroblasts [11], [13].

CD10/CALLA is a surface biomarker of MECs expressed during breast maturation. The expression of CD10 is decreased in DCIS and completely lost in invasive breast cancer [14]. We hypothesized that within the spectrum of DCIS, there is a continuum in CD10 expression, with lower values of CD10 expression reflecting loss of integrity of the basement membrane and, accordingly, an increased risk of local relapse. The aim of the present study was to retrospectively evaluate CD10 expression in DCIS tumors and to determine its association with long-term disease free survival (DFS).

## Materials and Methods

The present study adopted the REMARK guidelines for tumor markers prognostic evaluation [15].

### Patients and Sample Collection

The tissue samples used in this study were obtained from the Jules Bordet Institute (Brussels) and Antwerp University Hospital and approved for secondary use by the institutions' respective ethical committees, in compliance with the Belgian law of 19 December 2008 on human corporal material.

The expression levels of CD10 were assessed on a set of 11 normal breast tissue samples (median age of 52.3 years) obtained from reduction mammaplasty and used to define a cut-off for high expression of CD 10. The cut-off was corroborated with a set of 10 morphologically normal ducts associated with DCIS (median age of 53.0 years). Both of these tissue sets were obtained from Jules Bordet Institute.

The prognostic value of the CD10 marker was investigated in a consecutive series of 154 paraffin-embedded (FFPE) samples from patients treated surgically (lumpectomy or mastectomy) for ductal carcinoma *in situ* (DCIS) at the Jules Bordet Institute (n = 66; median age of 53.5 years), and at Antwerp University Hospital (n = 88; median age of 54 years). Patients with adjacent invasive carcinoma (invasion > = 1mm in the stroma surrounding the ducts) were excluded. 58% of low VPNI risk patients were treated by lumpectomy alone; 41% of intermediate VPNI risk patients received adjuvant radiotherapy following lumpectomy; and 81% of high-risk patients underwent mastectomy. Only 6 patients received tamoxifen treatment. The patient characteristics are described in table 1.

**Table 1:** 

		*Bordet DCIS pop (N = 66)*			*Antwerp DCIS pop (N = 88)*			P-val.*
**Patient and tumor characteristics**								
**Age at diagnosis (years)** **>60** **40–60** **<40** ***UK***		23394/			28528/			*0.601*
**Menopausal status** **Premenopausal** **Perimenopausal** **Postmenopausal** ***UK***		23367			2174218			*0.533*
**Disease-free survival (years)** **(range)**		6.95(0.25–14)			5.32(0.5–21)			*0.124*
**Event** **Yes** **No**		1056			1078			*0.823*
**Tumor size (mm)** **≤15** **16–40** **≥40** ***UK***		23171511			392920/			*0.726*
**Margin width (mm)** **≥ 10** **1–9** **<1** ***UK***		10103115			152548/			*0.558*
**Pathological class** **1** **2** **3** ***UK***		3558/			2922352			*3.7E-09*
**Comedo necrosis** **Present**Absent***UK***		19425			51352			*0.238*
**Estrogen receptor status** **Positive** **Negative** ***UK***		/			621610			*/*
**VNPI risk score** **Low (4–6)** **Intermediate (7–9)** **High (10–12)** ***UK***		1251921			214918/			*3.8E-04*
**Treatment** **Lumpectomy** **Lumpectomy + Radiation** **Mastectomy** ***UK***	**VNPI** : *UK*8013	Low100/	Inter988/	High3115/	Low10533	Inter11181010	High12150	*0.470*

**Legend:** ***UK*** = Unknown; * Wilcoxon (Mann-Whitney U-test.

### Updated VNPI 2003 assessment [16]

Tumor size, margin width, nuclear tumor grade (low grade versus high grade) and presence of comedo-necrosis were centrally reviewed for all patients by a single breast-experienced pathologist (DL), blinded to CD10 expression and long-term outcome. Patient age at diagnosis was included, according to the updated VNPI calculation requirements.

### Assessment of CD10 expression pattern by IHC

The pattern of CD10 expression was assessed by immuno-histochemistry (IHC) on FFPE slides using the standard immunoperoxidase method with an automated DAKO immunostainer (monoclonal CD10, clone 56C6, Novocastra, UK; dilution 1∶50, antigen retrieval in Decloak 5 min in TRS). Each immunohistochemical CD10 analysis was defined as a score combining the expression and intensity values (range from 2 to 6). CD10 expression was reported on a scale of 1 to 3 as focal ([1] 10% of duct circumference), partial ([2] 10%–90% of duct circumference), or circumferential ([3] >90% of duct circumference). The staining intensity was evaluated on a scale of 1 to 3, with 1 designated as weak staining, 2 as intermediate, and 3 as intense.

### Quantification of CD10 expression by qRT-PCR

Haematoxilin-eosin slides were also reviewed by a single breast-experienced pathologist (DL) charged with the task of circling the DCIS portion of the slide for tumor cell enrichment using TMA technology. RNA from FFPE samples was extracted from 8 punch biopsies (0.6 mm diameter and 2 mm sections) using the MasterPure™ Purification kit (Epicentre, Madison, WI) after paraffin removal with xylene. A DNase 1 treatment step was included. RNA was quantified using the NanoDrop® ND-1000 UV-Vis Spectrophotometer (NanoDrop Technologies, Wilmington, DE).

Reverse transcription (RT) was performed using a Super-Script™First-Strand Synthesis kit for qRT-PCR (Invitrogen Corp., Carlsbad, CA). Total RNA (300 ng) was reverse transcribed in a final volume of 21 µl with 50 ng of random hexamers. An RNase H treatment step was included.

Quantitative PCR reactions were performed in 96-well plates using Applied Biosystems ABI Prism® 7900HT (TaqMan® instruments). Gene expression was measured in duplicate using 5ng equivalent cDNA per reaction well. Amplifications were performed in 25 µl PCR mixture containing 300nM of each primer and 12,5 µl 2× SYBR® Green PCR Master Mix (Applied Biosystems). After 2 min at 50°C and 10 min at 95°C, cDNA was subjected to 40 cycles of PCR, with a denaturation step at 95°C for 30 sec followed by a combined annealing/extension step at 60°C for 1 min.

CD10 (NM_007289) forward primer (TGGGTTCTTGAAGGACATCTTTC) and reverse primer (CGTTACGGCAACTTTGACATTTT) were designed using the Primer Express® software (PE Applied Biosystems). Four housekeeper genes were selected on the basis of the literature as reference genes for data normalization (*TFRC*, *GUS*, *RPLPO* and *TBP*) (Table 2).

**Table 2:** Forward and reverse primers sequences for signature and normalization genes.

Gene Name	Accession number	Primer sequence
Genes of Normalization		
***GUS***	NM_000181	**-GAGTGGTGCTGAGGATTGGC** **-TCTAGCGTGTCGACCCCATT**
***TBP***	NM_003194	**-GCCCGAAACGCCGAATAT** **-TCGTGGCTCTCTTATCCTCATGA**
***RPLP0***	NM_001002	**-ACCAAGGAGGACCTCACTGAG** **-ACCAGCACGGGCAGCAG**
***TFRC***	NM_003234	**-GGAGCCAGGAGAGGACTTCC** **-TTCTCCGACAACTTTCTCTTCAGG**

### Data analysis

qRT-PCR data normalization: the average value of the expression of housekeeper genes was used as reference, and a threshold cycle (Ct) value was defined for each sample by taking off this average
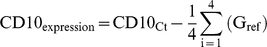
where CD10_expression_ is the CD10 threshold cycle and G_ref_ is the sum of the four housekeeper genes (TBP, RPLPo, GUS, TFRC).

All DCIS samples with expression levels lower than those of the normal breast samples were considered as CD10 low-expressing samples.

### Survival analysis

Disease free survival (DFS) was defined as either locoregional relapse of DCIS and/or the presence of invasive breast carcinoma. Non-breast cancer primaries and non-breast cancer deaths were considered as non-events. Survival curves were computed using the Kaplan-Meier product limit estimator. Hazard ratios for continuous and discrete variables were estimated through Cox's proportional hazard regression models.

Two-tailed p-values are reported, and p-values<0.05 were considered statistically significant. All statistical analyses were carried out using R version 2.5.1 and SPSS version 15 (SPSS Inc. 1999, Chicago, IL).

## Results

### Pattern of CD10 expression in normal breast tissue and DCIS

**Figure 1:**
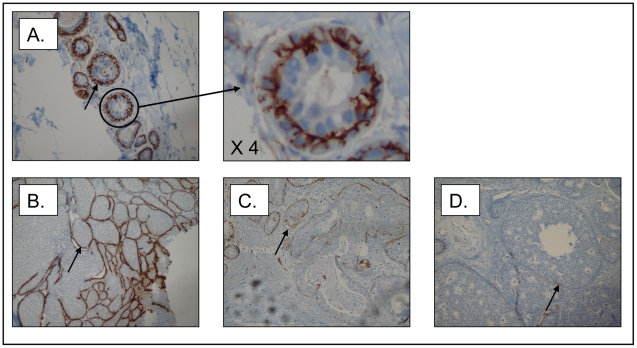
CD10 immunostaining of the myoepithelial cell (MECs) layer (original magnification **×**200). (A) intense staining (3) and a circumferential membrane expression (3) observed on normal breast (score = 6); (B) intense staining (3) and a circumferential membrane expression (3) observed on DCIS (score = 6); (C) intermediate staining (2) and partial membrane expression (2) observed on DCIS (score = 4); (D) weak staining (1) and focal membrane expression (1) observed on DCIS (score = 2).

In normal breast tissue (n = 11) and available DCIS tissue from the Jules Bordet Institute (n = 62/88), CD10 protein expression was exclusively localized on the MECs. Strong homogeneous membrane staining (score = 6, see Materials and Methods section) in the MECs was observed in all normal breast samples (figure 1.A). In contrast, DCIS showed a variable range of CD10 immunostaining. High CD10 immunostaining (score = 6), similar to that observed in the normal breast tissue, was present in 27% (17/62) of the DCIS samples, whereas 73% (45/62) showed CD10 immunostaining below the levels observed in the normal tissue (score<6) (figure 1.B and 1.C).

### Quantification of CD10 expression by qRT-PCR

**Figure 2:**
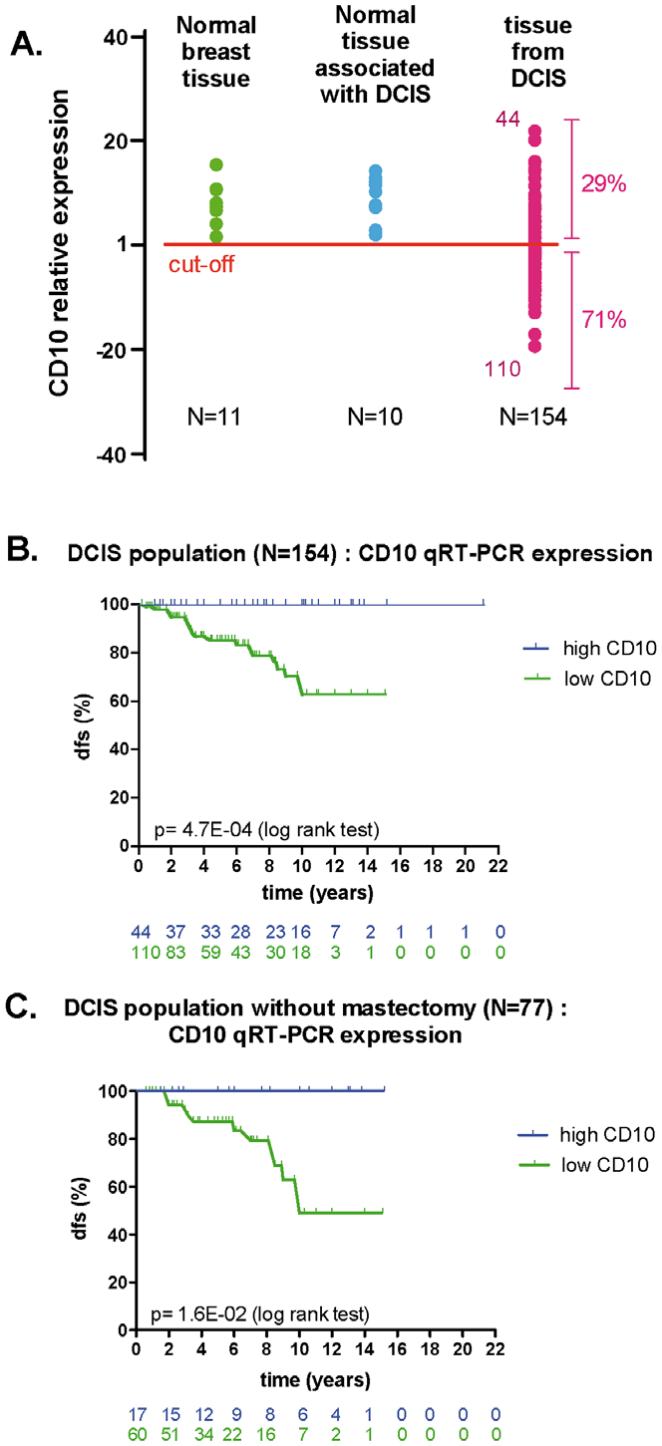
CD10 expression and clinical outcome. (A) scatter plot of CD10 expression evaluated by qRT-PCR for normal (N = 11) and DCIS (N = 154) samples. All normal samples and one-third of the DCIS were considered CD10 positive. (B) Kaplan-Meier curves of disease-free survival for patients with DCIS (N = 154) according to CD10 mRNA expression by qRT-PCR expression. (C) Kaplan-Meier curves of disease-free survival for patients with DCIS treated by tumorectomy only or tumorectomy and radiation (N = 77) according to CD10 mRNA expression by qRT-PCR expression.

CD10 mRNA level was quantified by qRT-PCR on FFPE samples from normal breast tissue (n = 11), normal breast associated with DCIS (n = 10) and DCIS samples (N = 154). A high CD10 mRNA level was reported for all normal breast tissue (n = 21) as well as 29% of the DCIS (figure 2.A), representing a proportion consistent with the one for CD10 immunostaining. DCIS samples were divided in two groups based on CD10 mRNA level: one group whose expression level was lower than that of the normal breast tissues (low CD10-expressing subgroup), and one group whose expression was similar to that of the normal samples (high CD10-expressing subgroup).

There was a significant but weak correlation between CD10 expression measured by IHC and qRT-PCR (ρ = 0.35, p = 5E-03). When both technologies were compared, CD10 status was discordant in 32% of the cases.

### CD10 expression and clinical outcome

CD10 mRNA expression was associated with long-term outcome in the 154 pure DCIS samples. Twenty (13%) loco-regional relapses (in situ or invasive) were documented after a median follow-up of 6years (range 0.25 to 21.1 years).

Low CD10 mRNA expression was statistically associated with increased risk of relapse (HR = 1.88; [95CI: 1.30–2.70], p = 0.001) when CD10 was considered as a continuous variable. Similar results were seen when DCIS patients were divided into low and high CD10 expressing subgroups (p = 4.7E-04). None of the 44 patients with high CD10 mRNA experienced a relapse (figure 2.B). The DFS at 10 years for the high CD10 group was 100% versus 63% for the low CD10 group. No prognostic value was found when CD10 expression was measured by IHC (log rank test p = 0.73; HR = 1.46; [95CI: 0.93–2.30], p = 9.5E-02).

Because patient treatment was heterogeneous (lumpectomy, lumpectomy and radiotherapy, mastectomy), we analyzed the association between the expression of CD10 and clinical outcome in each treatment subgroup separately. Low CD10 expression was associated with a 24%, 18.5% and 14% rate of relapse in the group of patients treated with lumpectomy, lumpectomy plus radiotherapy and mastectomy, respectively. In the groups of patients treated by lumpectomy or lumpectomy plus radiotherapy, none of the patients with high CD10 mRNA (N = 17/77) experienced a relapse (p = 1.6E-02) (figure 2.C). The DFS at 10 years for the high CD10 group was 100%, as compared to 41% for the low CD10 group.

### VNPI and clinical outcome

**Figure 3:**
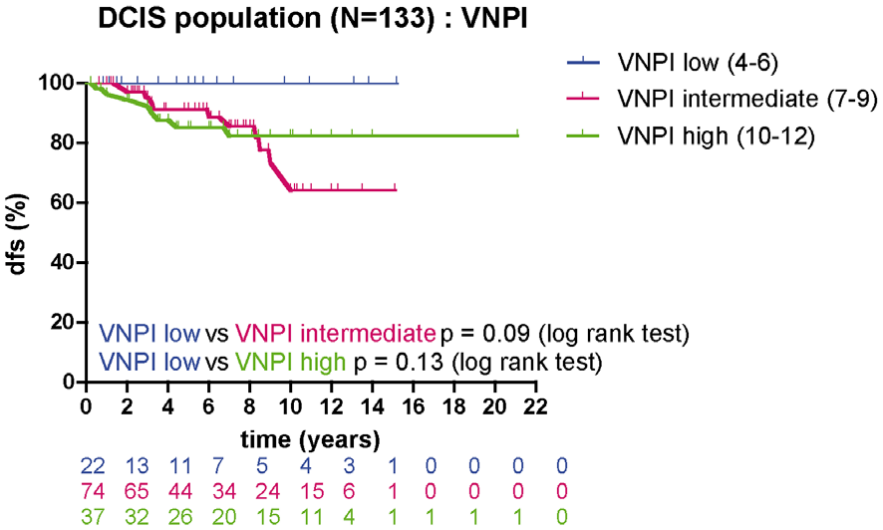
VNPI and clinical outcome: Disease-free survival analysis for patients with DCIS (N = 133) according to VNPI risk group.

Since the revised VNPI is considered to be a useful tool to predict local relapse in DCIS, we sought to investigate its prognostic performance in our population. The VNPI score was available for 86% (133/154) of the patients in this study: 16.5% (22/133) were low VNPI risk, 55.5% (74/133) were intermediate VNPI risk, and 28% (37/133) were high VNPI risk. The observed 10-year DFS was 100% for the low VPNI risk group, 64% for the intermediate VPNI risk group, and 84% for the high VNPI risk group (figure 3).

### Combined CD10 and VPNI risk

**Figure 4:**
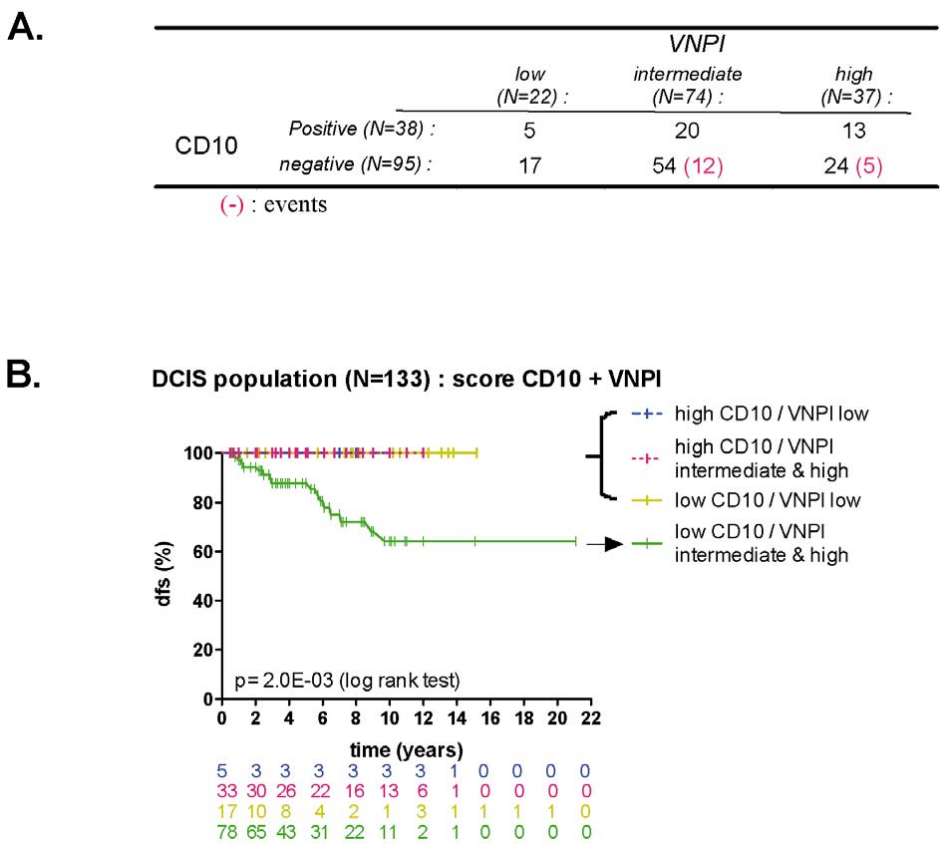
Combined CD10 and VPNI risk. (A) Cross-tabulation between CD10 qRT-PCR expression and VNPI score. (B) Disease-free survival analysis for patients with DCIS according to CD10 mRNA expression by qRT-PCR combined with VNPI risk group.

We evaluated the prognostic outcome according to combined VPNI and CD10 risk. Seventeen of the 22 patients (77%) considered as low VNPI risk had high CD10 risk (CD10 mRNA low), whereas 33 of the 38 patients (87%) with high CD10 mRNA expression (low CD10 risk) were considered intermediate or high VNPI risk (figure 4.A). A greater proportion of patients were considered low risk based upon CD10 expression (CD10 mRNA high) (28.5%, 38/133) when compared to VNPI (16.5%, 22/133). By combining both criteria, 41% (55/133) of the patients were reclassified as low risk group (figure 4.B). Of interest, none of those patients experienced a relapse (p = 1.6E-02).

A multivariate analysis demonstrated that both CD10 and VNPI were the only independent prognostic factors (HR_CD10_ = 2.39; [95%CI: 1.52–3.76], p = 0.0001; HR_VNPI_ = 1.39; [95%CI: 1.01–1.94], p = 0.05) when selecting CD10 mRNA level, VNPI score, treatments and menopausal status.

## Discussion

In our study, CD10 protein expression by IHC was located exclusively on the MECs in normal breast and in one-third of the DCIS, reflecting a well-organized MEC layer and a proportion between the one reported by Kalof *et al.* [14] and Hilson *et al.* [17]. However, in two-thirds of the DCIS samples lower CD10 expression was observed, suggesting an alteration of the MEC layer, which totally disappears in invasive breast cancer [14]. In some cases of invasive breast cancer, CD10 expression has been reported in the fibroblasts and associated with a bad prognosis, but this is a biological process independent from the one observed in this study.

CD10 mRNA expression was retrospectively evaluated using qRT-PCR, and its prognostic performance was assessed in a population of 154 DCIS paraffin-embedded samples. CD10 mRNA was expressed in all normal breast tissue and one-third of the DCIS patients, a proportion in the same range as our findings for IHC. Of clinical relevance, no relapse occurred in the group of DCIS patients with high CD10 expression. In contrast, low CD10 mRNA expression was significantly associated with an increased risk of relapse following resection of DCIS. A similar trend was found in the subgroup of patients treated with mastectomy alone. These findings support the notion that the progression of DCIS to invasive cancer may be due, in part, to MEC abnormalities that result in a loss of their normal tumor suppressor functions. Allinen *et al.* [18] reported that when compared with normal MECs, DCIS-associated MECs show down-regulation of a variety of genes involved in normal functions, including those for oxytocin receptor, laminin and thrombospondin, and up-regulation of genes for chemokines that enhance epithelial cell proliferation, migration, invasion and stromal angiogenesis, such as SDF1/CXCL12 and CXCL14. DCIS-associated MECs also showed increased levels of enzymes involved in the degradation of the extracellular matrix, such as matrix metalloproteinases, which might be responsible for local invasion.

In our cohort, the VNPI – considered a useful prognostic marker for treatment decision making in DCIS – could potentially avoid overtreatment in 16% of the DCIS patients; using CD10, this figure reached 28.5%. With respect to clinical relevance, both VNPI and CD10 were independent prognostic indicators of relapse. However, by combining both predictors we were able to identify a group of patients representing up to 41% of the DCIS patient population with no relapse at 10 years of follow up. All the patients with relapsing tumors were distributed into one of two groups: either a group with high VNPI scores representative of high tumor stage, or one with low CD10 expression reflecting at least some MEC layer alterations. Both characteristics – tumor stage and low CD10 expression – seem to be necessary for predicting relapse, which highlights the importance of assessing intrinsic DCIS properties as well as the surrounding microenvironment.

### Conclusion

Decreased expression of CD10 in DCIS is associated with a higher risk of local relapse. These promising results are currently being validated in a larger patient series. It is hoped that assessment of CD10 combined with VPNI may lead to improved treatment tailoring for women with DCIS in the future.
